# A Regime-Based Theoretical and Visualization Framework for Droplet Formation in Microfluidic Co-Flow Systems

**DOI:** 10.3390/mi17101117

**Published:** 2026-09-24

**Authors:** Van Thanh Hoang, Doan Hung Vo, Quang Hieu Tran, Anh Quan Tran, Minh Thong Tran, Quoc-Thong Le-Van, Jin-Wei Liang, Hsi-Hsun Tsai

**Affiliations:** 1 Department of Mechanical Engineering, The University of Danang—University of Science and Technology, 54 Nguyen Luong Bang Street, Danang City 550000, Vietnam; 2 Department of Mechanical Engineering, Incheon National University, Incheon 22012, Republic of Korea; 3 School of Mechanical Engineering, Chungnam National University, Daejeon 34134, Republic of Korea; 4 Department of Mechanical Engineering, Ming Chi University of Technology, New Taipei City 24301, Taiwan; 5 Research Center for Reliability Engineering, Ming Chi University of Technology, New Taipei City 24301, Taiwan; 6 Research Center for Intelligent Medical Devices, Ming Chi University of Technology, New Taipei City 24301, Taiwan

**Keywords:** microfluidics, co-flow stream, theoretical model, dripping regime, narrowing-jetting regime, widening jetting regime

## Abstract

Droplet formation in microfluidic co-flow systems is essential for producing controlled emulsions in biomedical, pharmaceutical, chemical, and materials applications. Despite extensive studies of a wide range of operating conditions, existing predictive and nondimensional relations are generally restricted to specific flow regimes and physical mechanisms. In this study, we develop a regime-based theoretical and visualization framework for predicting droplet generation across three representative co-flow regimes: dripping, narrowing jetting, and widening jetting. The proposed framework combines force-balance and mass-conservation principles to derive explicit analytical expressions for droplet size and droplet-generation frequency in terms of key dimensionless parameters, including the outer capillary number, viscosity ratio, and flow-rate ratio. For dripping, droplet size decreases with increasing outer capillary number. In narrowing jetting, droplet size decreases while generation frequency increases due to jet stretching and thinning. In widening jetting, droplet size increases with flow-rate ratio. In addition, the influence of the viscosity ratio weakens as the value increases. The models are validated against experimental data from the literature and show good agreement in predicting droplet size and frequency across multiple flow regimes and operating conditions. This work provides a physics-guided, regime-aware reduced-order framework for analyzing and designing microfluidic co-flow droplet-generation systems.

## 1. Introduction

Microfluidics has emerged as a key enabling technology in pharmaceutical, biomedical, and environmental engineering due to its ability to perform rapid analyses while significantly reducing the consumption of samples and reagents. Within this context, droplet generation in co-flowing streams plays a central role in a broad range of applications, including emulsification, controlled drug delivery, single-cell analysis, inkjet printing, and advanced material synthesis [1,2,3]. The ability to precisely control droplet size is particularly critical, as it directly influences transport phenomena, reaction kinetics, and the overall functional performance of microfluidic processes [4,5]. Microfluidic platforms form the technological foundation of Lab-on-a-Chip (LoC) and micro total analysis systems (microTAS), which enable the integration of multiple chemical and biological operations within micro devices [6,7,8,9]. Owing to the characteristic low Reynolds number (*Re*) conditions, flows in microfluidic systems are typically laminar, allowing highly controlled manipulation of fluids and reagents [10,11]. In addition, surface tension became dominant, leading to the rapid development of droplet-based microfluidics as a powerful strategy for compartmentalizing, transporting, and processing fluids at the microscale [12,13,14,15]. In these systems, droplets serve as discrete microreactors to control and manipulate the chemicals and reagents more precisely [16,17,18].

In droplet-based microfluidic systems, a range of droplet-generation techniques has been developed, each governed by distinct hydrodynamic mechanisms. Common configurations include T-junctions, flow-focusing junctions, step-emulsification modules, and co-flowing streams based on coaxial capillary arrangements [19,20,21,22,23]. In T-junction and flow-focusing geometries, droplet breakup is primarily induced by shear stresses imposed by the continuous phase at confined junctions, whereas step-emulsification relies on geometric expansion to trigger interfacial instability and droplet breakup [4,24]. Among these techniques, co-flowing stream configurations provide distinct advantages for fundamental and applied studies, as they allow independent control of the inner and outer fluid flow rates, viscosity contrast, and interfacial tension while maintaining a simple and axisymmetric geometry [25,26]. This flexibility makes co-flow systems particularly suitable for systematically investigating droplet deformation, breakup, and regime transitions under well-defined hydrodynamic conditions. Previous studies have shown that droplet formation in co-flowing streams is governed by multiple physical mechanisms that vary with key dimensionless parameters, including the flow-rate ratio, viscosity contrast, outer-phase capillary number, inner-phase Weber number, and Reynolds number in the low-Reynolds-number regime [11,25,27,28]. Early numerical investigations provided foundational insights into co-flow breakup dynamics by elucidating the effects of viscosity ratio, Bond number, and inner Capillary number on droplet formation under low-Reynolds-number conditions [29,30].

An experimental method was depicted to generate highly monodisperse emulsions. They reported that droplet size is a function of the velocity of the continuous phase, diameter of capillary tube, flow-rate ratios between dispersed phase and continuous phase, the viscosity and interfacial tension of the two phases [31]. A microcapillary device was used to produce monodisperse double emulsions, in which droplet size depends on the flow profile of the fluids [32]. Transition from dripping to jetting in co-flowing streams can be characterized by the outer capillary and the inner Weber number [33]. Effects of viscosity ratio, outer capillary number, and inner Weber number on the dynamics of dripping, widening jetting, and narrowing jetting in co-flowing fluid streams were investigated numerically and experimentally by Chen et al. (2013) [34]. Castro-Hernández et al. (2009) carried out an extensive experiment to investigate the dynamics of the jetting regime, including widening and narrowing [25]. They also proposed a linear model to predict droplet size, which is a function of jetting diameter.

For flow-focusing systems, Ibrahim et al. (2021) investigated the key parameters governing droplet formation at the flow-focusing junction, including the rate of change, the time at which the change starts, and the relative position of the dispersed phase at the nozzle [35]. They further developed a three-dimensional numerical model to evaluate how variations in flow rate influence the resulting droplet size. More recently, an improved correlation was developed linking droplet length, dimensionless flow rate, and the Weber number, achieving higher accuracy than traditional linear models [36]. L. He et al. (2025) extended these efforts by examining channels with different aspect ratios, demonstrating that the daughter-droplet length follows a power-law relationship with the outer capillary number (${Ca}_{o}$) at the second junction, and that the scaling exponent of the minimum neck thickness increases with the continuous-phase flow rate [28].

In the dripping regime, breakup occurs close to the nozzle, where the dispersed phase grows at the capillary tip and subsequently undergoes necking and pinch-off. The droplet size and breakup dynamics are governed by the coupled effects of the dispersed- and continuous-phase flow rates, viscosities, and interfacial tension [20]. As the outer-phase viscous stress becomes sufficiently strong, the dispersed phase is stretched into a slender downstream jet before breakup. This outer-phase-driven jetting regime is commonly referred to as narrowing jetting because the jet diameter decreases in the downstream direction. Its breakup is associated with the growth and convection of capillary disturbances along the jet and can be described in terms of Rayleigh–Plateau instability [25,33]. In contrast, when the inertia of the inner phase becomes sufficiently important, jetting can occur even at relatively low outer-phase capillary numbers. The resulting jet expands downstream, giving rise to widening jetting, for which the breakup dynamics differ from those of narrowing jetting and have been associated with absolute instability of the co-flowing jet [33].

Figure 1 illustrates the droplet-formation process of the flow emerging from the nozzle, including the dripping regime (Figure 1a), the narrowing-jetting regime (Figure 1b), and the widening jetting regime (Figure 1c). According to Chen et al. [34], when the inner-phase Weber number and viscosity ratio are fixed, a sharp change in droplet diameter occurs at a specific value of $Ca_{o}$, indicating the transition from dripping to narrowing-jetting. Therefore, the corresponding $Ca_{o}$ should be regarded as an approximate, system-specific transition threshold rather than a universal criterion, as illustrated in Figure 1b. Below this threshold, droplet breakup occurs in the dripping regime, whereas above it, the flow enters the narrowing-jetting regime under the corresponding experimental conditions. In contrast, widening jetting occurs when the forcing generated by the inner phase becomes sufficiently strong, and its onset is strongly influenced by the inner-phase Reynolds number, $Re_{i}$ [25,33].

Despite extensive numerical and experimental investigations of droplet-formation dynamics in co-flow systems, the development of predictive theoretical models for regime behavior remains relatively limited. Existing models are often restricted to individual breakup regimes or narrow operating windows, which reduces their usefulness for broader microfluidic design and optimization. In particular, a consistent analytical framework capable of describing droplet formation across the dripping, narrowing jetting, and widening jetting regimes in co-flowing streams has remained limited. Motivated by this gap, the present study develops regime-based theoretical models to describe droplet deformation and breakup behavior in co-flow systems. For each regime, droplet size is characterized using key dimensionless parameters, especially the outer capillary number and the viscosity ratio, which govern the competition between viscous and interfacial forces, together with the flow-rate ratio where required. Explicit relationships are established between these governing parameters and the dominant physical mechanisms controlling breakup, thereby providing a predictive basis over a broad range of operating conditions. In addition to the analytical development, this study also introduces a visualization framework based directly on the derived theoretical expressions. While analytical models provide quantitative predictions of droplet size and generation frequency, they offer limited intuitive representation of the droplet-formation process itself. The proposed visualization framework addresses this limitation by translating the regime-dependent analytical results into a graphical representation of the co-flow geometry, droplet evolution, breakup mode, and droplet spacing. In this way, the present work extends beyond numerical prediction alone and provides a more physically interpretable framework for analyzing droplet behavior in microfluidic co-flow systems.

The geometry of the co-flow system is described in Section 2. The theoretical models for the three droplet-generation regimes are presented in Section 3. The corresponding results and discussion are provided in Section 4, while Section 5 presents the droplet-generation visualization framework. Finally, Section 6 concludes the paper with a summary of the main findings and recommendations.

## 2. Geometry Description

The co-flow system is made of two cylindrical capillary tubes which are aligned coaxially as shown in Figure 1. The diameters of the inner capillary tube and outer capillary tube are d_i_ and d_o,_ respectively. The surface tension between the inner liquid and outer liquid is denoted by *σ*. λ is the viscosity ratio between the inner fluid and outer fluid $\left(\mu_{i}/\mu_{o}\right)$. The density ratio of the inner fluid to the outer fluid is unity. The dispersed phase is injected with a flow rate of *q_i_* into a continuous phase which flows with a flow rate of *q_o_*.

The medium and the dispersed phase are assumed to be incompressible Newtonian liquids, and the flow is assumed to be laminar. A volume of fluid model is used to capture the interface between the droplet and medium [16], and the continuum surface force model is used to handle the surface tension as a body force [37].

Figure 2 illustrates the flow configuration for a co-flowing stream system, which is used to establish the theoretical framework for droplet formation and to clarify the roles of the governing forces and parameters. The model considers a two-dimensional, axisymmetric, nonlinear flow, in which a dispersed liquid is injected through an inner nozzle into a reservoir containing a continuous liquid flowing concurrently [38].

Among the forces acting on the dispersed phase, the surface-tension force $F_{s}$ and the viscous drag force $F_{d}$ represent the dominant contributions in the present reduced-order model. The surface-tension force acts as a capillary-retention force, tending to minimize the interfacial area and resist droplet elongation and detachment from the nozzle. In contrast, the viscous drag force exerted by the continuous phase acts downstream and promotes deformation, necking, and eventual breakup of the dispersed phase. The inner-phase inertial force $F_{i}$ may also contribute to downstream deformation, although it remains small under the dripping conditions considered here and becomes increasingly important toward the widening-jetting regime. Gravitational and buoyancy forces, $F_{g}$ and $F_{b}$, are negligible because of the small characteristic droplet size and the resulting low Bond number. In addition, the hydrodynamic effects of the continuous phase are represented collectively through the effective drag force $F_{d}$, while the capillary contribution is represented by $F_{s}$; therefore, local pressure and interfacial-stress components are not introduced separately in the present reduced-order formulation.

## 3. Proposed Models

### 3.1. Forces Balance Approach

In co-flowing devices, droplet generation occurs in the dripping regime when breakup takes place near the nozzle tip under conditions where both the outer-fluid viscous stress and the inner-fluid inertia remain weak relative to interfacial tension. In this regime, droplet detachment is commonly interpreted as a balance between the drag exerted by the continuous phase and the capillary force retaining the dispersed phase at the tip. As the flow conditions increase beyond a critical threshold, the breakup mode transitions from dripping to jetting, in which a liquid filament forms before pinching off downstream, as schematically illustrated in Figure 2, which shows the co-flow configuration and the characteristic forces considered in the present force-balance model. Depending on the relative importance of outer-phase viscous shear and inner-phase inertia, the jetting behavior may appear as narrowing jetting or widening jetting [20]. Following Utada et al. [33], the dripping-to-jetting transition in co-flowing streams can be characterized by the outer-phase capillary number $Ca_{o}$ and the inner-phase Weber number $We_{i}$, with the transition occurring when $Ca_{o}+We_{i}\;\sim \;O(1)$.

At sufficiently high inner-phase flow rates, inertial effects become comparable to capillary forces, and the maximum flow rate before sustained jet formation can therefore be estimated from an inertia–capillarity balance as:

(1) $$\;q_{i}^{max}\sim \sqrt{\frac{\gamma d_{i}^{3}}{\rho_{i}},}$$

where γ is the interfacial tension, $d_{i}$ is the inner nozzle diameter, and $\rho_{i}$ is the inner-phase density.

Several forces may contribute to droplet dynamics in a co-flowing channel, including viscous drag, surface tension, buoyancy, Laplace pressure, and momentum-transfer effects; however, under the present low-Reynolds-number microfluidic conditions, viscous drag and surface tension forces are assumed to be dominant [39,40]. Under this assumption, droplet breakup occurs when the viscous drag force becomes comparable to or exceeds the capillary force that holds the droplet at the outlet of the dispersed-phase tube. Buoyancy is neglected because the characteristic droplet size is small, making gravitational effects weak relative to interfacial tension. The momentum-transfer term is neglected because the inner-phase inertia is assumed to remain small. The Laplace-pressure contribution is not introduced separately, since its capillary effect is already represented in the surface-tension holding force adopted in the model. Accordingly, the surface-tension force is approximated as:


(2) $$\;F_{s}=\pi d_{i}\gamma .$$

The viscous drag force is approximated using a modified Stokes-type expression as:

(3) $$\;F_{d}=3\pi \mu_{o}\left(d-d_{i}\right)\left(v_{o}-v_{i}\right)\frac{3\lambda +2}{A_{1}\lambda +A_{2}},$$

where d is the droplet diameter, $v_{o}$ is the mean velocity of the continuous phase, and $v_{i}$ is the characteristic downstream velocity of the inner phase. The characteristic downstream velocity of the dispersed phase is estimated from the volumetric flow rate, and the characteristic cross-sectional area of the forming droplet is expressed as $v_{i}=4q_{i}/\pi d^{2}$. In Equation (3), the factor $Y=\left(3\lambda +2\right)/\left(A_{1}\lambda +A_{2}\right)$ is introduced as a modified Hadamard–Rybczynski-type correction factor to represent the influence of the viscosity ratio on the viscous drag acting on the growing droplet. The classical Hadamard–Rybczynski relation is derived for a freely moving spherical droplet, whereas in the present dripping configuration the dispersed phase remains attached to the nozzle and undergoes continuous deformation before detachment. Therefore, $A_{1}$ and $A_{2}$ are introduced as fitting parameters to modify the viscosity-ratio dependence of the classical correction and account, in an effective manner, for the attached and deforming droplet configuration.

A droplet will be generated as long as the viscous drag force is larger than the surface-tension force. At the critical point, we have the resultant force condition as follows by applying the force-balance conditions, $F_{d}=F_{s}$. Substituting the retention force of Equation (2) and the drag force of Equation (3) into the detachment condition $F_{d}=F_{s}$ gives:


(4) $$\;3\pi \mu_{o}\left(d-d_{i}\right)\left(v_{o}-v_{i}\right)Y=\pi d_{i}\gamma .$$

Introducing the normalized droplet diameter $x=d/d_{i}$, such that $d=xd_{i}$, $d-d_{i}=d_{i}\left(x-1\right)$, and $d^{2}=x^{2}d_{i}^{2}$, converts Equation (4) into a dimensionless form. After multiplying by $x^{2}$, expanding the resulting expression, and collecting terms with equal powers of x, the cubic relation in Equation (5) is obtained.


(5) $$\;{\left(\frac{d}{d_{i}}\right)}^{3}-\left(\frac{1}{Y}\frac{\gamma }{3\mu_{o}v_{o}}+1\right){\left(\frac{d}{d_{i}}\right)}^{2}-\frac{4q_{i}}{\pi v_{o}d_{i}^{2}}\frac{d}{d_{i}}+\frac{4q_{i}}{\pi v_{o}d_{i}^{2}}=0.$$

A reduced model is obtained when considering $q_{i}$ is so small, so we have the corresponding result as follows:


(6) $$\;\frac{d}{d_{i}}=\frac{1}{Y}\frac{\gamma }{3\mu_{o}v_{o}}+1.$$

Equation (6) can be re-expressed in the form of Equation (7) by introducing $Ca_{o}=\mu_{o}v_{o}/\gamma$. In this reformulation, the factor 1/3 is absorbed into the fitted coefficients $A_{1}$ and $A_{2}$, while the constant term in Equation (6) is replaced by an additional fitting parameter B, yielding:


(7) $$\;\frac{d}{d_{i}}=\frac{A_{1}\lambda +A_{2}}{3\lambda +2}\frac{1}{{Ca}_{o}}+B.$$

For the narrowing-jetting regime, the droplet is no longer attached to the nozzle tip but instead forms through the breakup of a slender liquid thread. Therefore, the force-balance relation developed for the dripping regime cannot be applied directly without modification. Accordingly, Equation (7) is reformulated using the viscosity-ratio correction factor $\left(\lambda +1\right)/\left(3\lambda +2\right)$ [8], while two fitting parameters, C and D, are introduced to account for the different breakup characteristics of the narrowing-jetting regime, yielding Equation (8).


(8) $$\;\frac{d}{d_{i}}=\frac{\lambda +1}{3\lambda +2}\frac{C}{{Ca}_{o}}+D.$$

### 3.2. Mass Conservation Approach

In the classical picture proposed by Utada et al., narrowing jetting is triggered when the outer-phase capillary number becomes of order unity, $Ca_{o}∼O(1)$, whereas widening jetting is associated with the inner-phase Weber number becoming of order unity, $We_{i}∼O(1)$ [33]. Thus, when $Ca_{o}$ becomes sufficiently large, the dripping regime transitions to narrowing jetting, and a slender liquid thread forms and subsequently breaks into droplets through Rayleigh–Plateau instability [25,33]. By contrast, when the inner-phase forcing becomes dominant, the system transitions from dripping to widening jetting. Nevertheless, for the widening regime, Castro-Hernández et al. showed that the criterion $We_{i}>1$ is not universally applicable [25]. Specifically, they demonstrated that this condition is valid only when the inner-phase Reynolds number also satisfies $Re_{i}>1$. When $Re_{i}<1$, inertial effects are no longer the appropriate measure of the transition, and the dripping-to-widening-jetting criterion is better described by the inner-phase capillary number, $Ca_{i}$. Accordingly, widening jetting can be expected when $We_{i}≳1$ for $Re_{i}>1$, but when $Re_{i}<1$, the appropriate criterion becomes $Ca_{i}≳1$. This refinement indicates that widening jets may be driven either by inner-phase inertia or by inner-phase viscous stresses, depending on the magnitude of $Re_{i}$.

Under conditions where the outer flow rate exceeds the inner flow rate, the viscous stress imposed by the continuous phase generally dominates over inner-phase inertial stress, and droplet breakup can still be interpreted using a force balance between viscous drag and surface tension. However, when the inner flow rate becomes sufficiently large relative to the outer flow rate, the inner-phase stress becomes dominant over the viscous drag force. In this case, the net force balance between viscous drag and capillary force is no longer adequate to describe droplet breakup in the widening jetting regime. Instead, a mass-conservation-based approach, as expressed in Equation (9), can be employed to characterize droplet breakup in the widening jetting regime and to establish the scaling relationship governing droplet formation in this flow condition [27,41]. Following [41] and [32], the droplet pinch-off time is assumed to satisfy Equation (10).

(9) $$\;q_{i}=\frac{\pi d^{3}}{6}\frac{1}{t_{pinch\;}}.$$

(10) $$\;t_{pinch}=\frac{Cd_{jet}\mu_{o}}{\gamma },$$

where $d_{jet}$ is the jet diameter, and C is a coefficient that depends on the viscosity ratio. However, C should not be regarded as a universal constant. Since the present reduced formulation does not explicitly resolve the full viscosity-ratio dependence of the breakup dynamics, C is treated here as a viscosity-ratio-dependent closure term. To preserve a compact analytical form, this dependence is represented using the power-law relation $C(\lambda )=K\lambda^{m}$, where K and m are fitting constants. Accordingly, the pinch-off timescale is rewritten as:


(11) $$\;t_{pinch}=\frac{K{\lambda^{m}d}_{jet}\mu_{o}}{\gamma }.$$

Substituting $t_{pinch}$ from Equation (11) into the mass-conservation equation in Equation (9), and using the relation $d_{jet}=d_{o}\sqrt{X/(X+1)}$, where $X=\lambda \left(\sqrt{1+q_{i}/(q_{o}\lambda )}-1\right)$ [42], after algebraic manipulation, the relationship between the normalized droplet diameter $\left(d/d_{o}\right)$ and the flow-rate ratio $\left(q_{i}/q_{o}\right)$ in the widening jetting regime can be expressed as:


(12) $$\;\frac{d}{d_{o}}=K{\left(\lambda^{m}\frac{q_{i}}{q_{o}}{Ca}_{o}\sqrt{\frac{X}{X+1}}\right)}^{1/3}.$$

### 3.3. Droplet-Generation Frequency

Under steady operating conditions in the dripping and narrowing-jetting regimes, the dispersed phase is injected into the co-flowing system at a constant volumetric flow rate, $q_{i}$. The droplet-formation process is assumed to be periodic and repeatable, and mass loss of the dispersed phase is neglected. Accordingly, the droplet-generation dynamics can be analyzed using a mass-conservation approach over a characteristic time interval, Δt, following previous treatments of droplet-based microfluidic systems [25,34]. During this interval, the total volume of dispersed phase entering the system is given by $\Delta V_{i}=q_{i}\Delta t$.

Over the same interval, the number of generated droplets is N=fΔt, where f($s^{-1}$) denotes the droplet-generation frequency, defined as the average number of droplets produced per unit time [34]. The total volume of droplets formed during this interval is therefore $\Delta V_{b}=NV_{b}=f\Delta tV_{b}$, where $V_{b}$ is the volume of an individual droplet. By enforcing mass conservation, $q_{i}\Delta t=f\Delta tV_{b}$, which leads to the general expression:


(13) $$\;f=\frac{q_{i}}{V_{b}}.$$

For spherical droplets, the droplet volume is given by $V_{b}={\pi d}^{3}/6$. In the dripping and narrowing-jetting regimes, the droplet size is primarily governed by the balance between the viscous stresses imposed by the continuous phase and interfacial tension. This balance is characterized by the outer capillary number, $Ca_{o}$, which represents the relative importance of viscous forces to surface tension [25,29].

Based on the force-balance formulation, substituting the normalized droplet diameter from Equation (7) and Equation (8) into the expression for droplet volume yields Equation (14) for the dripping regime and Equation (15) for the narrowing-jetting regime, respectively.


(14) $$\;V_{b}=\frac{\pi }{6}{d_{i}^{3}\left(\frac{A_{1}\lambda +A_{2}}{3\lambda +2}\frac{1}{{Ca}_{o}}+B\right)}^{3}$$

(15) $$\;V_{b}=\frac{\pi }{6}{d_{i}^{3}\left(\frac{\lambda +1}{3\lambda +2}\frac{C}{{Ca}_{o}}+D\right)}^{3}$$

Substituting Equations (14) and (15) into Equation (13) and performing the algebraic operations yields Equations (16) and (17) for dripping ${(f}_{d})$ regime and narrowing-jetting regime ${(f}_{n})$, respectively, as follows:


(16) $$\;f_{d}=\frac{6q_{i}}{\pi d_{i}^{3}}{\left(\frac{A_{1}\lambda +A_{2}}{3\lambda +2}\frac{1}{{Ca}_{o}}+B\right)}^{-3}$$

(17) $$\;f_{n}=\frac{6q_{i}}{\pi d_{i}^{3}}{\left(\frac{\lambda +1}{3\lambda +2}\frac{C}{{Ca}_{o}}+D\right)}^{-3}$$

## 4. Results and Discussion

The theoretical prediction model for the transition from the dripping regime to the narrowing-jetting regime, and subsequently to the widening jetting regime, is presented in the next subsection. The relationship between the characteristic parameters, which depend on the different flow types, is described through fitting constants. These models are then compared with previous experimental results and studies to assess the accuracy of the theoretical model proposed by the authors.

### 4.1. The Proposed Model for Dripping Regime

For the dripping regime, where $A_{1}$, $A_{2}$, and B are fitting constants, these parameters were determined by fitting Equation (7) to the experimental data reported by Chen et al. [34] as a function of $Ca_{o}$. The resulting fitted values are $A_{1}=0.51683$, $A_{2}=0.70076$, and B=0.96279. Figure 3 presents the performance of the proposed droplet-size model for the dripping regime under different operating conditions. As shown in Figure 3a, the data of Chen et al. [34] were first used for parameter identification over the range $Ca_{o}$ from 0.08 to 0.20, within which the normalized droplet diameter decreases from approximately $d/d_{i}=3.6$ to about 1.7. The calibrated model was then compared with the independent experimental results of Umbanhowar et al. [31], as shown in Figure 3b, for λ=3.3. Taken together, these results indicate that the proposed expression successfully captures the dominant droplet-size scaling in the dripping regime. Overall, the model achieved a coefficient of determination of $R^{2}=0.986$, confirming its high predictive accuracy.

Figure 3c presents the predicted variation in normalized droplet size as a function of the $Ca_{o}$ for a wide range of λ. For all λ, the droplet size decreases monotonically as $Ca_{o}$ increases, with the strongest reduction occurring in the low- $Ca_{o}$ region. For example, when λ=0.1, $d/d_{i}$ decreases from roughly 7.3 at $Ca_{o}=0.05$ to about 2.0 at $Ca_{o}=0.30$, while for λ=10, it decreases from about 4.7 to approximately 1.6 over the same range. This trend indicates that increasing $Ca_{o}$ intensifies the viscous stress exerted by the continuous phase relative to interfacial tension, thereby enhancing the elongation of the dispersed phase and accelerating pinch-off. As a result, the residence time available for volumetric accumulation at the nozzle tip is shortened, leading to progressively smaller droplets. Another important feature is that the difference in droplet size among different λ values becomes less pronounced as $Ca_{o}$ increases. At low $Ca_{o}$, the spread between the λ=0.1 and λ=20 curves is large, on the order of about three droplet diameters in normalized form, whereas at $Ca_{o}=0.30$, this difference shrinks to less than about 0.5. In particular, for λ=10 and 20, the prediction curves nearly overlap over most of the plotted range, indicating that once the dispersed phase becomes sufficiently more viscous than the continuous phase, the effect of further increasing λ is weak and the droplet size becomes governed predominantly by $Ca_{o}$.

Figure 3d further examines the influence of viscosity ratio on droplet size. First, the model is again compared with the experimental results at $Ca_{o}=0.113$, showing good agreement and thus providing additional validation of the proposed formulation. The same figure also presents the predicted variation of $d/d_{i}$ as a function of λ for several fixed $Ca_{o}$ values. For all cases, the droplet size decreases sharply in the low- λ region and then gradually approaches a plateau as λ increases. At $Ca_{o}=0.113$, for instance, the model predicts a decrease in $d/d_{i}$ from approximately 4.0 at very small λ to about 2.7 at λ=20, with most of this reduction occurring below λ≈5. Similarly, at $Ca_{o}=0.2$, $d/d_{i}$ drops from about 2.6 to around 1.9, and at $Ca_{o}=0.3$, it decreases from roughly 2.0 to about 1.6. This behavior indicates that, in the low- λ regime, increasing the dispersed-phase viscosity substantially modifies the interfacial deformation and suppresses droplet growth before detachment, thereby producing smaller droplets. The saturation of droplet size with increasing viscosity ratio in Figure 3d follows directly from Equation (7). The viscosity-ratio dependence is contained in the bounded factor $\left(A_{1}\lambda +A_{2}\right)/\left(3\lambda +2\right)$, which approaches $A_{2}/2$ as λ→0 and $A_{1}/3$ as λ→∞. With the fitted constants, these limits are 0.350 and 0.172, respectively, restricting the total variation to approximately a factor of two. Physically, this behavior is consistent with the Hadamard–Rybczynski mobility: increasing λ progressively suppresses internal droplet circulation until the hydrodynamic response approaches the rigid-particle limit. Further increases in λ then produce only minor changes, leading naturally to the observed saturation.

### 4.2. The Proposed Model for Narrowing-Jetting Regime

For the narrowing-jetting regime, the fitting constants C and D were determined by fitting Equation (8) to the experimental data reported by Chen et al. [34]. The fitting procedure yielded C=2.168917 and D=−0.569536, with an overall coefficient of determination of $R^{2}=0.986$, indicating excellent agreement between the proposed relation and the experimental observations. Figure 4 summarizes the performance of the resulting model for droplet-size prediction in the narrowing-jetting regime. As shown in Figure 4a, the predicted normalized droplet diameter is compared with the experimental data for λ=10, showing good agreement over the considered range of $Ca_{o}$. In particular, both the model and experiments show a strong reduction in droplet size from approximately $d/d_{i}=1.8$ at $Ca_{o}=0.3$ to about $d/d_{i}=0.2$ at $Ca_{o}=1.0$, confirming that the proposed expression successfully captures the dominant scaling behavior in the narrowing-jetting regime.

Figure 4a also presents the predicted variation in normalized droplet size as a function of $Ca_{o}$ for different λ values. In all cases, the droplet size decreases monotonically with increasing $Ca_{o}$, and the reduction is more pronounced than in the dripping regime because breakup now occurs from a slender liquid jet rather than from direct accumulation at the nozzle tip. As $Ca_{o}$ increases, the viscous stress imposed by the outer phase intensifies, leading to stronger jet stretching and thinning before pinch-off, which in turn reduces the detached droplet volume. Quantitatively, at $Ca_{o}=0.2$, the predicted droplet size varies from about $d/d_{i}=4.6$ for λ=0.1 to about $d/d_{i}=3.1$ for λ=20, whereas at $Ca_{o}=1.0$, all curves collapse into a much narrower range, approximately $d/d_{i}\approx 0.2$ to 0.5. This indicates that the influence of λ becomes progressively weaker as $Ca_{o}$ increases. At a fixed $Ca_{o}$, increasing λ leads to smaller droplets, especially in the low- λ range, because a more viscous dispersed phase modifies the jet profile and promotes the formation of a thinner, more stable liquid thread prior to breakup.

Figure 4b further illustrates the influence of viscosity ratio by plotting the predicted droplet size as a function of λ for several fixed values of $Ca_{o}$. For all three cases, $d/d_{i}$ decreases rapidly at low λ and then gradually approaches an asymptotic value as λ increases. For example, at $Ca_{o}=0.3$, the normalized droplet size decreases from about 3.0 at very small λ to approximately 1.9 at λ=20, with most of this change occurring below λ≈5. Similarly, at $Ca_{o}=0.6$, $d/d_{i}$ drops from around 1.2 to about 0.65, while at $Ca_{o}=0.9$, it decreases from roughly 0.6 to about 0.25. Another notable feature is that, at a fixed λ, the difference in droplet size between $Ca_{o}=0.3$ and 0.6 is substantially larger than that between $Ca_{o}=0.6$ and 0.9. This indicates that the sensitivity of droplet size to $Ca_{o}$ becomes weaker at higher $Ca_{o}$, where the jet is already strongly stretched, and further increases in outer-phase viscous stress produce only limited additional thinning. Overall, these results suggest that in the narrowing-jetting regime, $Ca_{o}$ still remains the dominant parameter controlling droplet size, while λ exerts a secondary but still noticeable influence, particularly in the low- λ region.

To further assess the regime-specific nature of the proposed formulations, a cross-validation analysis is provided in Appendix A (Figure A1). The results show that each model accurately represents its corresponding regime but exhibits clear deviations when applied to data from the other regime, supporting the use of separate formulations for dripping and narrowing jetting.

### 4.3. The Proposed Model for Widening Jetting Regime

For the widening jetting regime, the fitting constants K and m in Equation (12) were determined using the experimental data reported by Castro-Hernández et al. [25], yielding K=10.65095 and m=0.427616. The predictive performance of the resulting model is assessed in Figure 5a, where the calculated normalized droplet diameter, $d/d_{o}$, is compared with the experimental results for λ=0.1, 1, and 10. Overall, the model reproduces the experimental trends with reasonable accuracy, with an overall coefficient of determination of $R^{2}=0.875$. The agreement is particularly good for the lower viscosity ratios, λ=0.1 and 1, where the model closely follows the increase of $d/d_{o}$ with $q_{i}/q_{o}$. For λ=10, the agreement remains relatively good, although the deviation becomes larger than in the lower- λ cases, especially at the smallest flow-rate ratios. These results confirm that the proposed scaling relation is capable of describing the primary droplet size in the widening jetting regime.

Figure 5b further illustrates the predicted dependence of droplet size on the flow-rate ratio $q_{i}/q_{o}$ for a broader range of viscosity ratios, λ=0.01, 0.1, 1, 5, and 10. In all cases, the normalized droplet size increases monotonically with increasing $q_{i}/q_{o}$, indicating that a stronger inner-phase throughput relative to the outer-phase flow produces a thicker jet and therefore a larger detached droplet volume. The increase is nonlinear, with a steeper slope in the low- $q_{i}/q_{o}$ region and a more gradual rise at higher $q_{i}/q_{o}$. At a fixed $q_{i}/q_{o}$, the droplet size also increases markedly with λ. For example, at $q_{i}/q_{o}=0.02$, the predicted $d/d_{o}$ increases from 0.16 for λ=0.01 to about 0.23 for λ=0.1, 0.32 for λ=1, 0.4 for λ=5, and nearly 0.45 for λ=10. A similar ordering is maintained throughout the plotted range. Physically, this trend indicates that a more viscous dispersed phase resists radial contraction more strongly and sustains a thicker liquid core before pinch-off, leading to larger droplets in the widening regime. Another notable feature is that the separation between the curves is more pronounced at low and intermediate λ, especially between λ=0.01 and 0.1, between λ=0.1 and 1, and between λ=1 and 5, whereas the incremental increase from λ=5  to 10 is smaller. This suggests that the influence of viscosity ratio remains strong over the low-to-intermediate range but gradually weakens as λ becomes large, reflecting a tendency toward partial saturation of viscosity-ratio effects in widening jet breakup.

### 4.4. Droplet-Generation Frequency in the Dripping and Narrowing-Jetting Regime

For the dripping-regime frequency model, the fitting constants $A_{1}$, $A_{2}$, and B in Equation (16) were identified by fitting the model to the experimental data reported by Chen et al. [34], yielding $A_{1}=0.271027$, $A_{2}=0.282469$, and B=0.3443. Figure 6 validates the resulting model against the experimental data under two conditions: at λ=10 in Figure 6a and at $Ca_{o}=0.113$ in Figure 6b, with an overall coefficient of determination of $R^{2}=0.991$, indicating excellent agreement between the proposed expression and the experimental observations.

Figure 6 also reveals clear parametric trends in the effects of $Ca_{o}$ and λ on droplet-generation frequency. As shown in Figure 6a, for all viscosity ratios, the frequency increases monotonically and nonlinearly with $Ca_{o}$, with the rise becoming especially pronounced at moderate and high $Ca_{o}$. For example, at $Ca_{o}=0.30$, the predicted frequency is about $3.5\times 10^{3}s^{-1}$ for λ=0.1, $4.8\times 10^{3}s^{-1}$ for λ=1, and approximately $6.3\times 10^{3}s^{-1}$ for λ=10 and 20. This behavior is physically consistent with the force-balance picture in the dripping regime: increasing $Ca_{o}$ strengthens the viscous stress imposed by the continuous phase relative to interfacial tension, thereby accelerating neck thinning and droplet detachment, which shortens the droplet-formation period and increases the generation frequency. Moreover, at very low $Ca_{o}$, the frequency curves corresponding to different λ values remain close to one another, suggesting that the effect of viscosity ratio is weak when capillary retention dominates over outer-phase viscous forcing.

Figure 6b shows the complementary effect of viscosity ratio. At fixed $Ca_{o}$, the frequency increases rapidly in the low- λ region and then gradually approaches a plateau as λ becomes large. At $Ca_{o}=0.113$, for instance, the frequency increases from about $0.5\times 10^{3}s^{-1}$ at very small λ to roughly $1.1\times 10^{3}s^{-1}$ at λ=20, with the Chen et al. data showing the same saturation-type trend. Similarly, at $Ca_{o}=0.2$ and 0.3, the asymptotic frequencies approach about $3.4\times 10^{3}$ and $6.4\times 10^{3}s^{-1}$, respectively. The much larger separation between the $Ca_{o}=0.113$, 0.2, and 0.3 curves compared with the variation along each individual curve indicates that $Ca_{o}$ is the dominant parameter controlling frequency, whereas the effect of λ is secondary and becomes progressively weaker at high λ. Physically, this suggests that once the dispersed-phase viscous response is sufficiently strong, further increases in λ only marginally affect the droplet volume and pinch-off timescale, so the frequency becomes governed primarily by the outer-phase viscous forcing represented by $Ca_{o}$.

For the narrowing-jetting frequency model, the fitting constants C and D in Equation (17) were determined by fitting the proposed relation to the experimental data [34], yielding C=0.777651 and D=0.015151, with an overall coefficient of determination of $R^{2}=0.9795$. As shown in Figure 7a, the model reproduces the experimental trend well for λ=10, confirming that the proposed expression can capture the main scaling of droplet-generation frequency in the narrowing-jetting regime. Similar to the dripping regime, the frequency increases monotonically with increasing $Ca_{o}$, but its magnitude is substantially larger because breakup now occurs from a continuously stretched and thinned liquid jet rather than from droplet growth directly at the nozzle tip. At $Ca_{o}=1.1$, the predicted frequencies reach approximately $40\times 10^{3}$, $67\times 10^{3}$, $102\times 10^{3}$, and $107\times 10^{3}s^{-1}$ for λ=0.1, 1, 10, and 20, respectively, clearly demonstrating the strong amplification of breakup rate in the narrowing-jetting regime. Figure 7b further shows the predicted variation in frequency with λ for $Ca_{o}=0.3$, 0.6, and 0.9. At $Ca_{o}=0.3$, the frequency remains relatively low, around $2\times 10^{3}s^{-1}$, over most of the λ range, whereas at larger $Ca_{o}$ values, the dependence on λ becomes much more pronounced. In particular, for $Ca_{o}=0.6$ and 0.9, the frequency increases sharply as λ rises up to about λ≈5, after which the increase becomes negligible, and the curves approach a plateau, with asymptotic values of about $18\times 10^{3}$ and $60\times 10^{3}s^{-1}$, respectively. Overall, these results indicate that $Ca_{o}$ remains the dominant parameter controlling frequency in the narrowing-jetting regime, while λ exerts a secondary but non-negligible influence, particularly before the viscosity-ratio effect saturates.

It should be noted that additional independent experimental validation was possible for the dripping regime using data reported in the literature. However, suitable datasets for the narrowing-jetting regime and droplet-generation frequency remain limited because many previous studies do not provide the complete set of parameters required by the present model. Further independent validation of these regimes is therefore still needed.

## 5. Droplet-Generation Visualization

The primary objective of this study is to develop theoretical predictive models for droplet formation in co-flow systems. After this work, without relying on experiments or numerical simulations, the behavior of droplet formation in a co-flow system could be predicted. However, although analytical models can effectively predict characteristic quantities such as droplet size and generation frequency, they are limited in their ability to immediately provide intuitive visual representations of the droplet-formation process. This raises an important question: can analytical expressions be further utilized to visually reconstruct the co-flow system and the corresponding droplet-generation behavior? To address this question, a visualization framework is presented in this section. The proposed framework uses the developed analytical models to represent the co-flow configuration and to visualize key droplet-formation features, including droplet size, breakup mode, and generation frequency. In this way, the framework extends the utility of the theoretical model beyond quantitative prediction alone, enabling a more intuitive interpretation of droplet-generation behavior under different operating conditions.

The droplet-generation visualization framework is implemented as an interactive browser-based interface using HTML, CSS, and JavaScript, with the droplet-formation process rendered in real time on an HTML5 canvas. The interface allows the user to specify the key geometric, fluid, and operating parameters of the co-flow system, including the inner nozzle diameter $d_{i}$, outer tube diameter $d_{o}$, viscosity ratio λ, outer capillary number $Ca_{o}$, and the inner and outer flow rates $q_{i}$ and $q_{o}$. The current implementation presents the three breakup modes (dripping, narrowing jetting, and widening jetting) through dedicated regime tabs and updates the predicted quantities immediately as the input parameters are modified.

The visualization is driven by regime-dependent theoretical expressions embedded in the computational module. For the dripping regime, the droplet diameter is evaluated using the fitted force-balance relation $d/d_{i}=f(\lambda ,Ca_{o})$ given in Equation (7), and the droplet-generation frequency is subsequently calculated from the mass-conservation-based expression in Equation (15). For the narrowing-jetting regime, a separate fitted diameter relation, given by Equation (8), is employed together with its corresponding frequency model in Equation (16). For the widening jetting regime, the droplet size is evaluated from a scaling relation expressed in terms of $d/d_{o}$, which depends on λ, $q_{i}/q_{o}$, $Ca_{o}$, and the auxiliary variable X. Since no dedicated frequency model for the widening jetting regime is proposed in the present study, the visualization in this regime is focused primarily on droplet-size behavior. An alternative frequency expression adopted from the dripping regime is used only to support the animation process and does not carry physical significance for widening jetting. The predicted droplet size and spacing are then passed to the animation routine. These computed quantities are subsequently mapped into screen coordinates through a geometric scaling procedure, enabling the nozzle, outer tube, jet, droplet growth, pinch-off, and downstream transport to be visualized in a physically interpretable manner. The necking and pinch-off shapes in the narrowing- and widening-jetting regimes are included for illustrative purposes and are not intended to quantitatively reproduce the local interface evolution.

Figure 8, Figure 9 and Figure 10 present visual comparisons between the proposed analytical visualization framework and reference results from the literature for the three droplet-generation regimes. Because the underlying analytical models have been validated against experimental data in previous subsections, the visualized results are consistent with the reference observations in terms of droplet size and droplet spacing (generation frequency). Figure 8 compares the visualization of the dripping regime with the experimental image reported by Chen et al. [34] showing that the predicted droplet shape, size, and spacing are consistent with the observed periodic droplet formation near the nozzle tip. Figure 9 illustrates the narrowing-jetting regime and is compared with the simulated results, which are included because this regime often exhibits droplet-size instability and more complex jet evolution than dripping. Despite these inherent fluctuations, the present visualization reproduces the overall characteristics of a slender jet followed by downstream droplet formation, while the detailed necking morphology is represented only schematically. Figure 10 presents the widening jetting regime in comparison with the experimental results of Castro-Hernández et al. [25], demonstrating that the analytical visualization can also capture the characteristic increase in droplet size associated with stronger inner-phase forcing.

## 6. Conclusions

This study proposed a regime-based analytical framework for droplet formation in microfluidic co-flow systems, covering the dripping, narrowing jetting, and widening jetting regimes. By combining force-balance and mass-conservation considerations, separate predictive expressions were established to describe droplet size and, where applicable, droplet-generation frequency in terms of the governing dimensionless parameters, including the outer capillary number, viscosity ratio, and flow-rate ratio. The use of separate formulations for different regimes was found to be necessary, since the breakup mechanisms differ substantially between tip-based dripping, outer-shear-dominated narrowing jetting, and inner-phase-driven widening jetting.

Comparison with representative experimental data from the literature showed that the proposed models can reasonably reproduce the main trends of droplet size and generation frequency over the considered operating ranges. In particular, the results confirmed that $Ca_{o}$ plays a dominant role in controlling droplet size and frequency in the dripping and narrowing-jetting regimes, while the effects of viscosity ratio become weaker in some high- $Ca_{o}$ conditions. For the widening jetting regime, the droplet-size scaling captured the overall dependence on flow-rate ratio and viscosity ratio, although the agreement was less strong than that of the dripping and narrowing-jetting cases. These results suggest that the proposed framework is useful for interpreting the dominant physical factors governing breakup behavior in co-flow systems.

The present framework should, however, be regarded as a physics-guided reduced-order model rather than a complete description of the two-phase flow field. Instead, the model reduces the complex two-phase behavior to the dominant force-balance and mass-conservation mechanisms relevant to each regime. Therefore, its predictions are most appropriately used within the validated operating ranges for rapid parametric assessment, preliminary design, and interpretation, while high-fidelity interface-resolved simulations remain necessary when detailed local flow and interfacial dynamics are required. In addition, the present study does not establish universal critical conditions for the transitions among dripping, narrowing jetting, and widening jetting. Future work should therefore focus on establishing more general regime-transition criteria.

In addition, a visualization framework was developed based on the analytical expressions, allowing regime-dependent droplet-formation behavior to be displayed in an intuitive manner. This component extends the analytical framework beyond quantitative prediction by providing a clearer representation of droplet evolution under different operating conditions. Overall, the proposed approach provides a simplified analytical and visualization framework that may serve as a computationally efficient tool for preliminary analysis, interpretation, and low-cost design support in microfluidic co-flow droplet-generation systems.

## Figures and Tables

**Figure 1:**
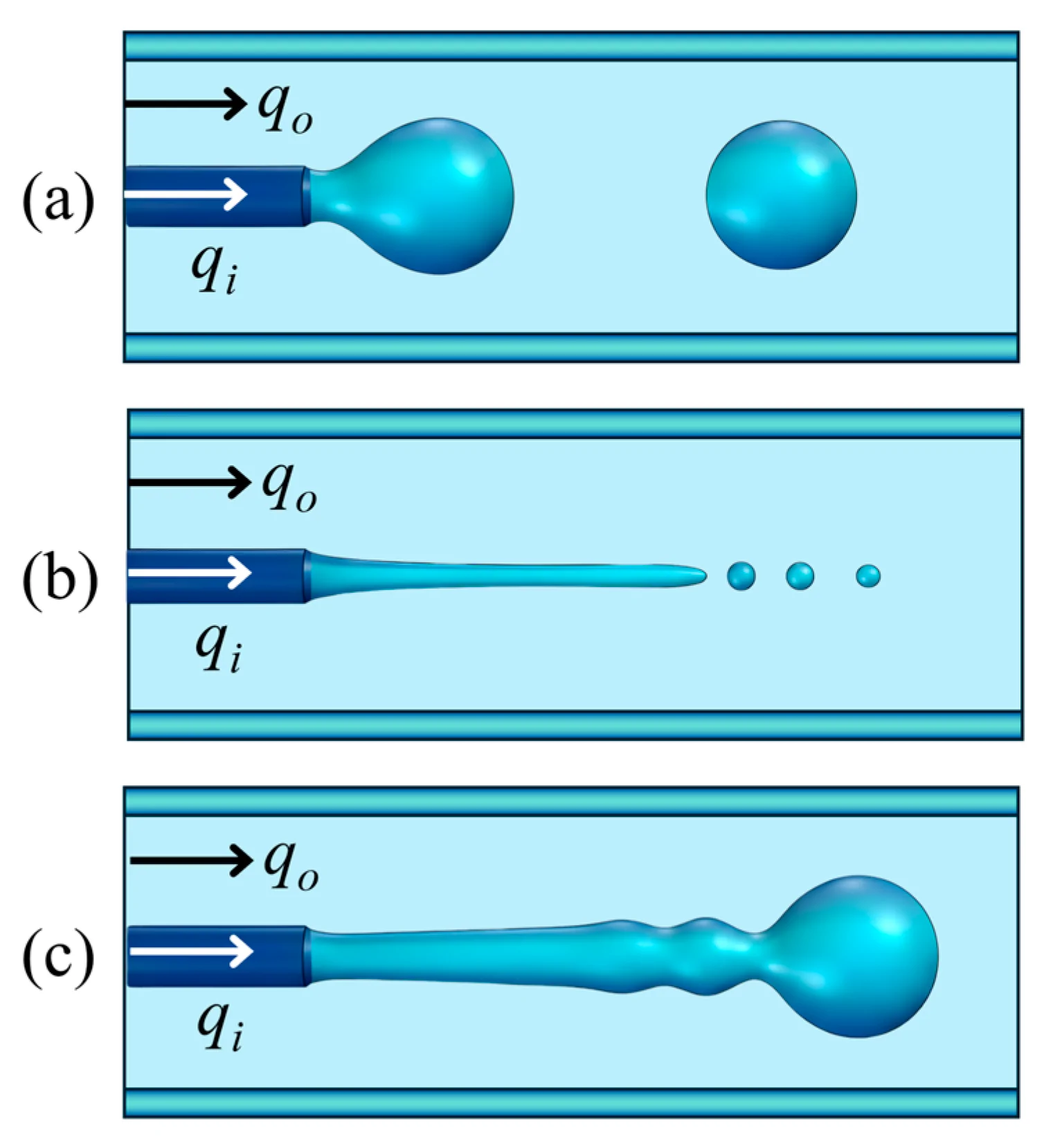
Dynamics of different regimes in co-flow stream: (**a**) dripping regime; (**b**) narrowing-jetting regime; (**c**) widening jetting regime.

**Figure 2:**
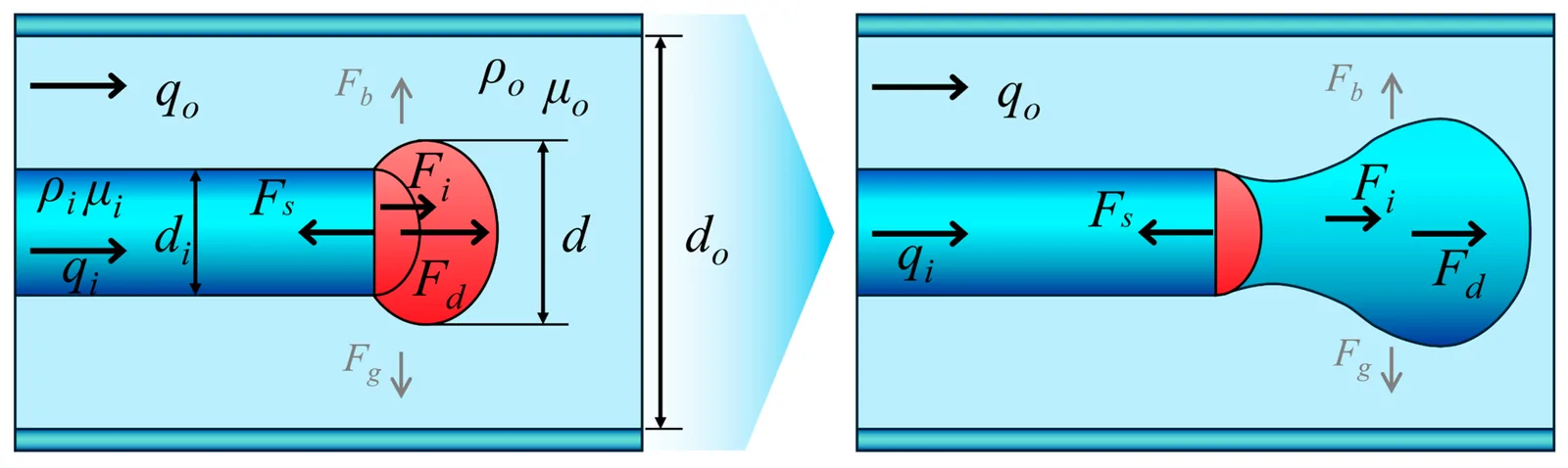
Schematic of the co-flow droplet-formation configuration and governing forces.

**Figure 3:**
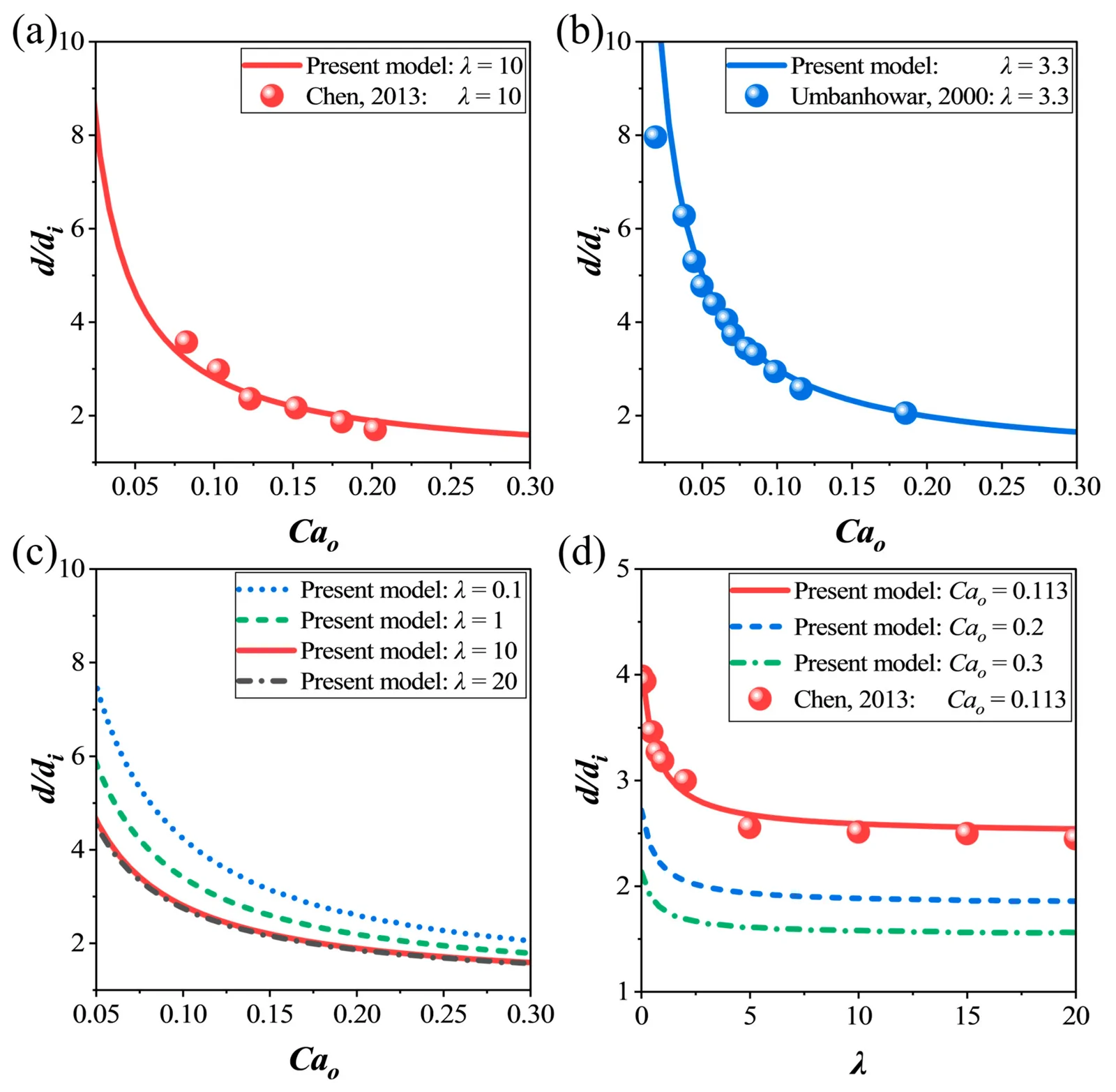
Droplet size for dripping regime under different conditions: (**a**,**b**) validation of the present model against experimental results reported by Chen et al. [34] and Umbanhowar [31]; (**c**) predicted variation in normalized droplet size, as a function of $Ca_{o}$ for different λ; (**d**) comparison of the present model with the experimental results of Chen et al. [34], along with the predicted variation in the normalized droplet size, as a function of λ for different values of $Ca_{o}$.

**Figure 4:**
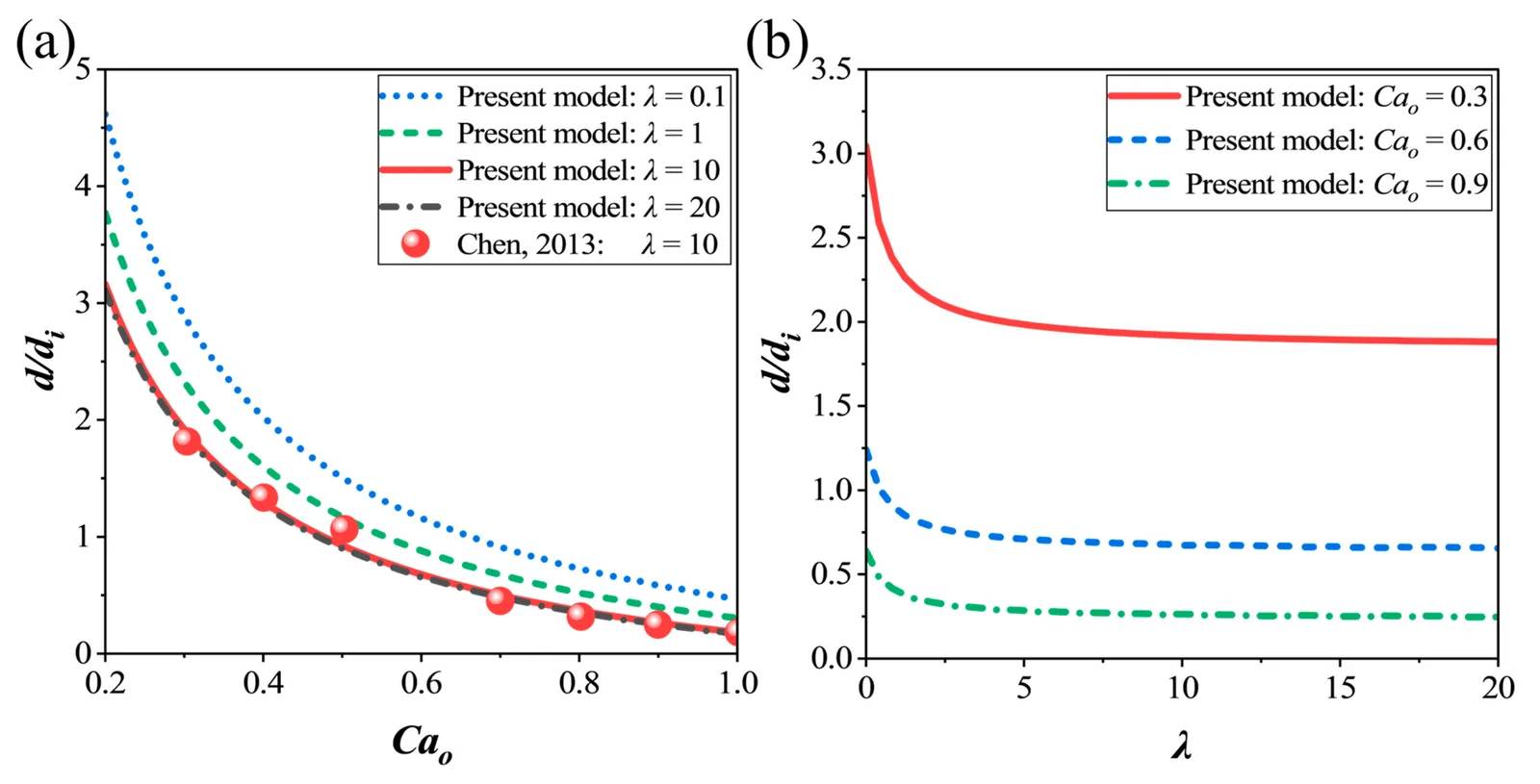
Droplet size in the narrowing-jetting regime under different conditions: (**a**) comparison of the present model with experimental data from Chen et al. [34], together with the predicted variation in the normalized droplet size as a function of $Ca_{o}$ for different λ; (**b**) predicted variation in the normalized droplet size as a function of λ for different values of $Ca_{o}$.

**Figure 5:**
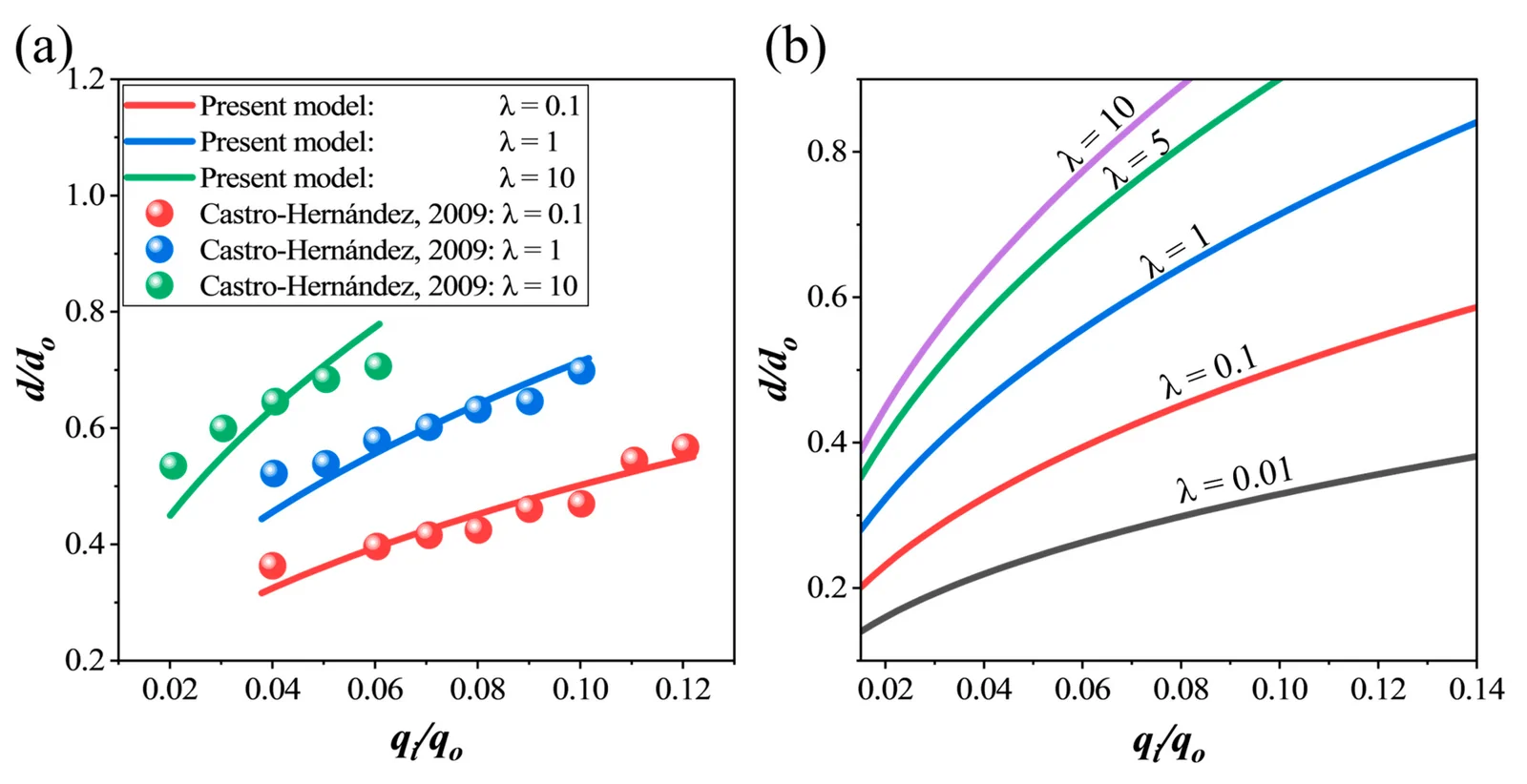
Droplet size predictions in the widening jetting regime under various operating conditions: (**a**) comparison of the present model with the experimental data of Castro-Hernández et al. [25] for different λ; (**b**) predicted variation in the normalized droplet diameter, $d/d_{o}$, as a function of the flow-rate ratio, $q_{i}/q_{o}$, for different values of λ.

**Figure 6:**
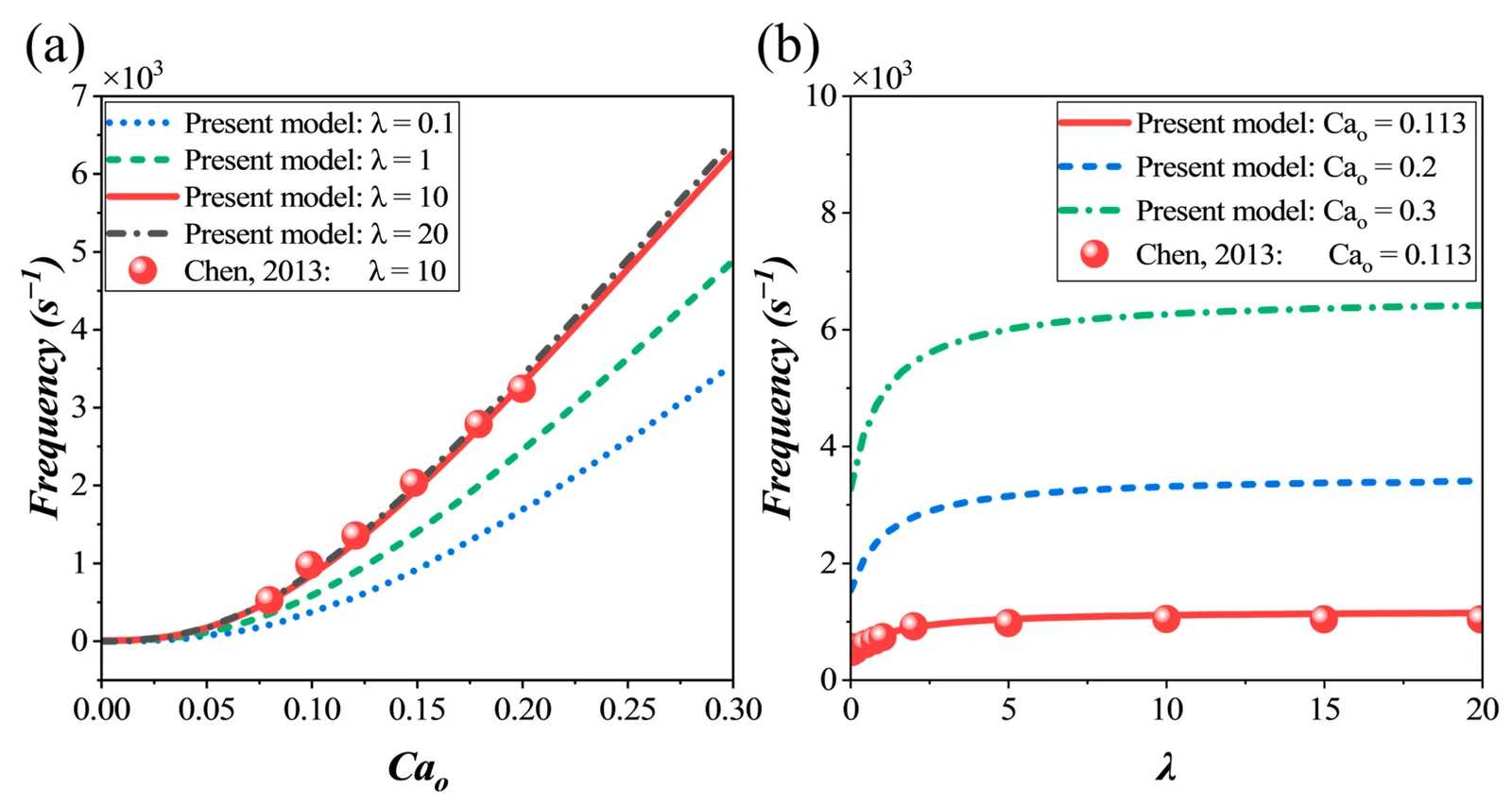
Droplet-generation frequency in the dripping regime under different conditions: (**a**) predicted variation in frequency as a function of $Ca_{o}$ for different λ; (**b**) predicted variation in frequency as a function of λ for different values of $Ca_{o}$ [34].

**Figure 7:**
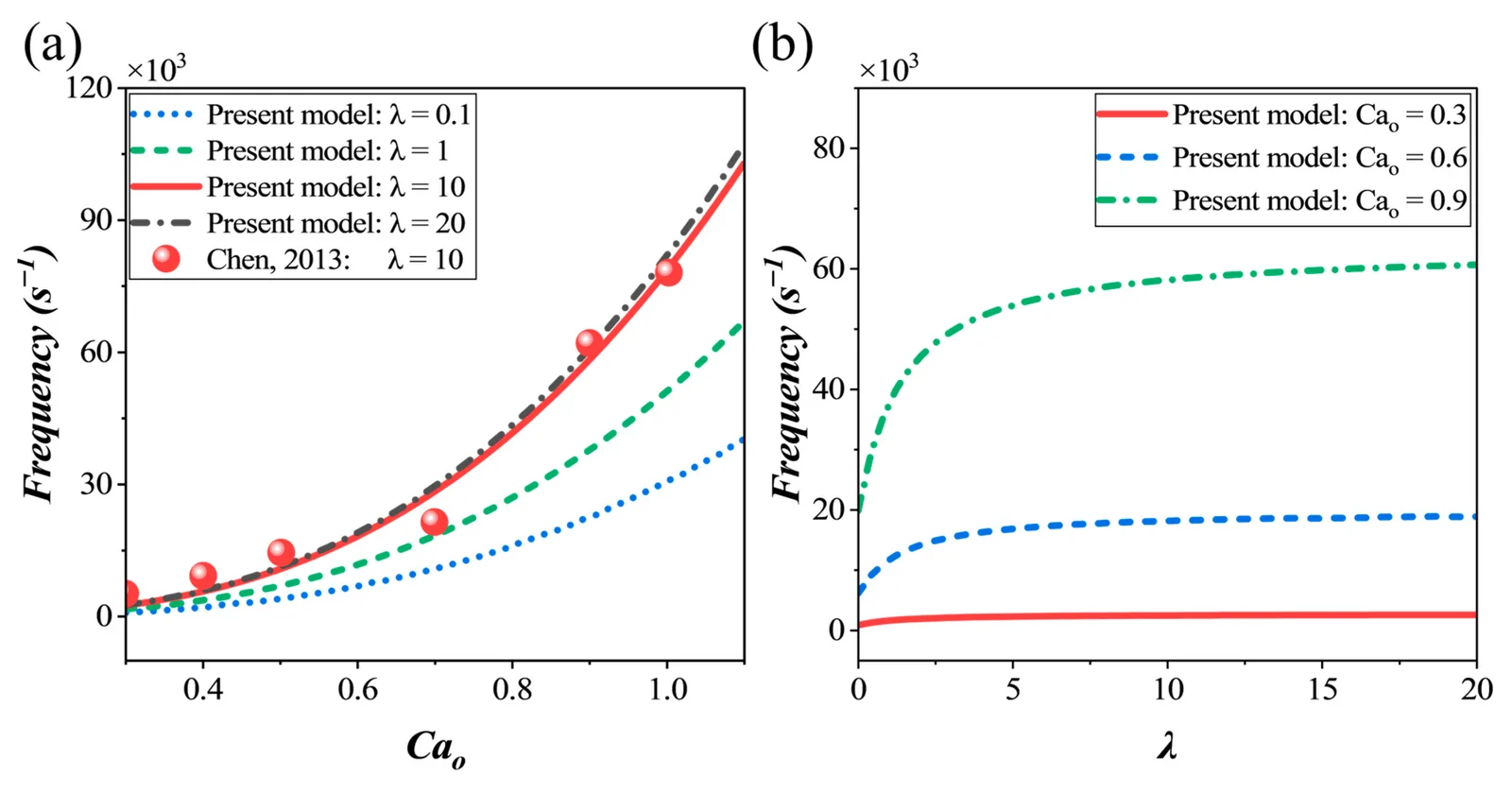
Droplet-generation frequency in the narrowing-jetting regime under different conditions: (**a**) comparison of the present model with the experimental data [34], together with the predicted variation in frequency as a function of $Ca_{o}$, for different λ; (**b**) predicted variation in frequency as a function of λ for different values of $Ca_{o}$.

**Figure 8:**
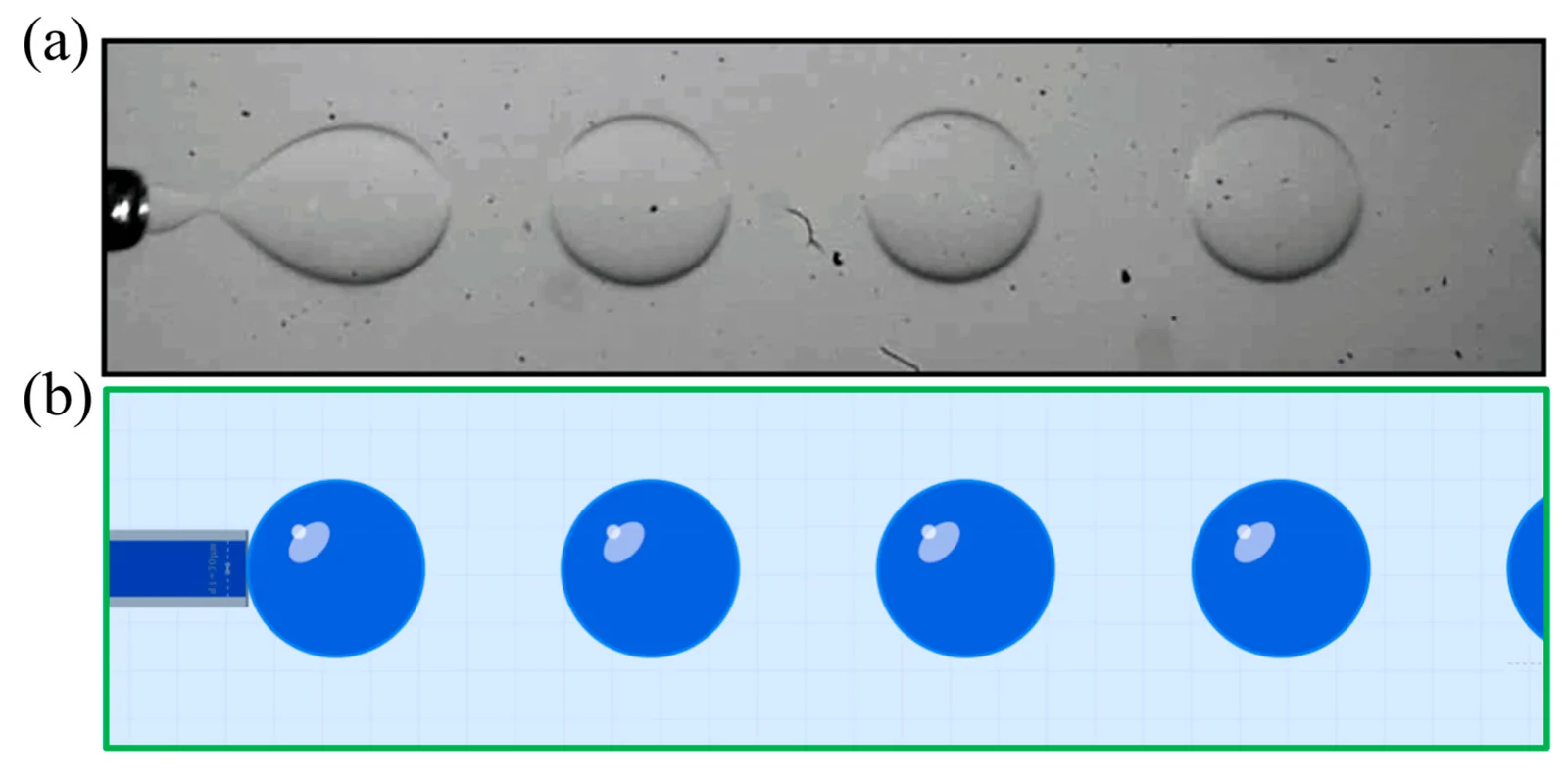
Comparison between the present visualization framework and the experimental results of Chen et al. [34] for the dripping regime: (**a**) experimental image (reproduced with permission); (**b**) corresponding visualization generated using the present analytical framework.

**Figure 9:**
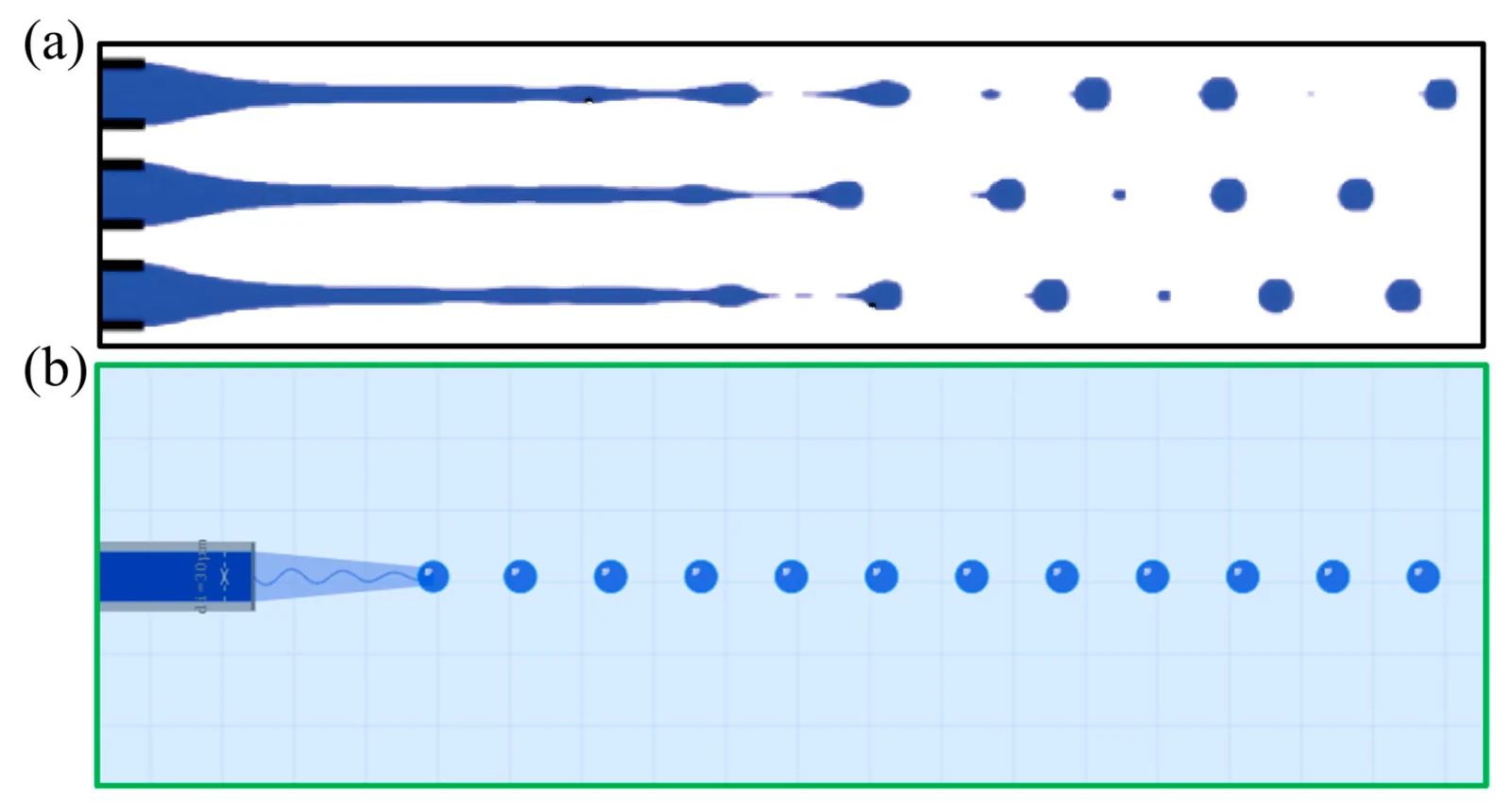
Comparison between the present visualization framework and the simulation results of Chen et al. [34] for the narrowing-jetting regime: (**a**) simulation images (reproduced with permission); (**b**) corresponding visualization generated using the present analytical framework.

**Figure 10:**
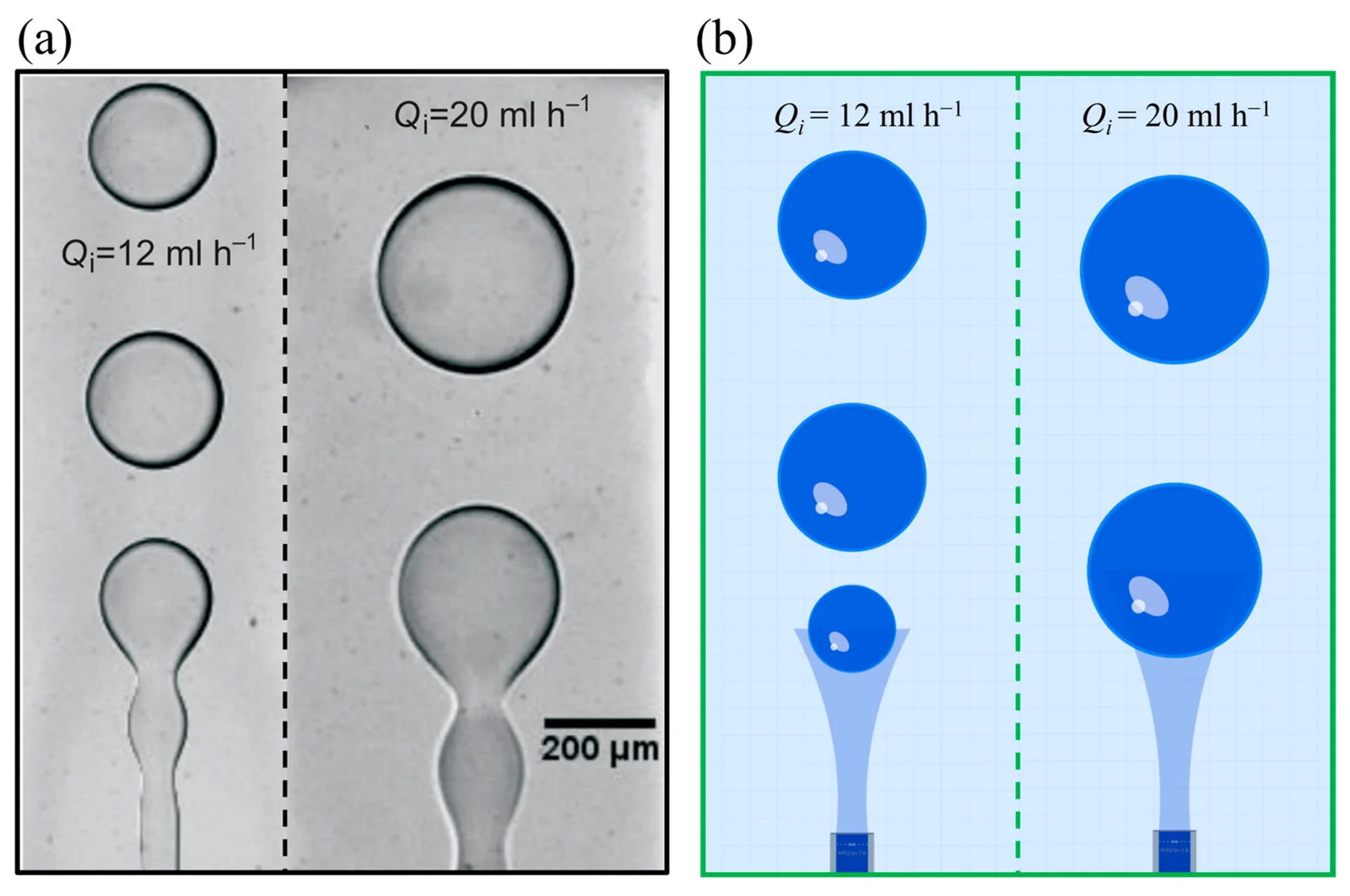
Visual comparison of the widening jetting regime between the experimental results of Castro-Hernández et al. [25] and the present analytical visualization framework: (**a**) experimental images (© Deutsche Physikalische Gesellschaft. Reproduced by permission of IOP Publishing. CC BY-NC-SA.); (**b**) present visualization.

**Figure A1:**
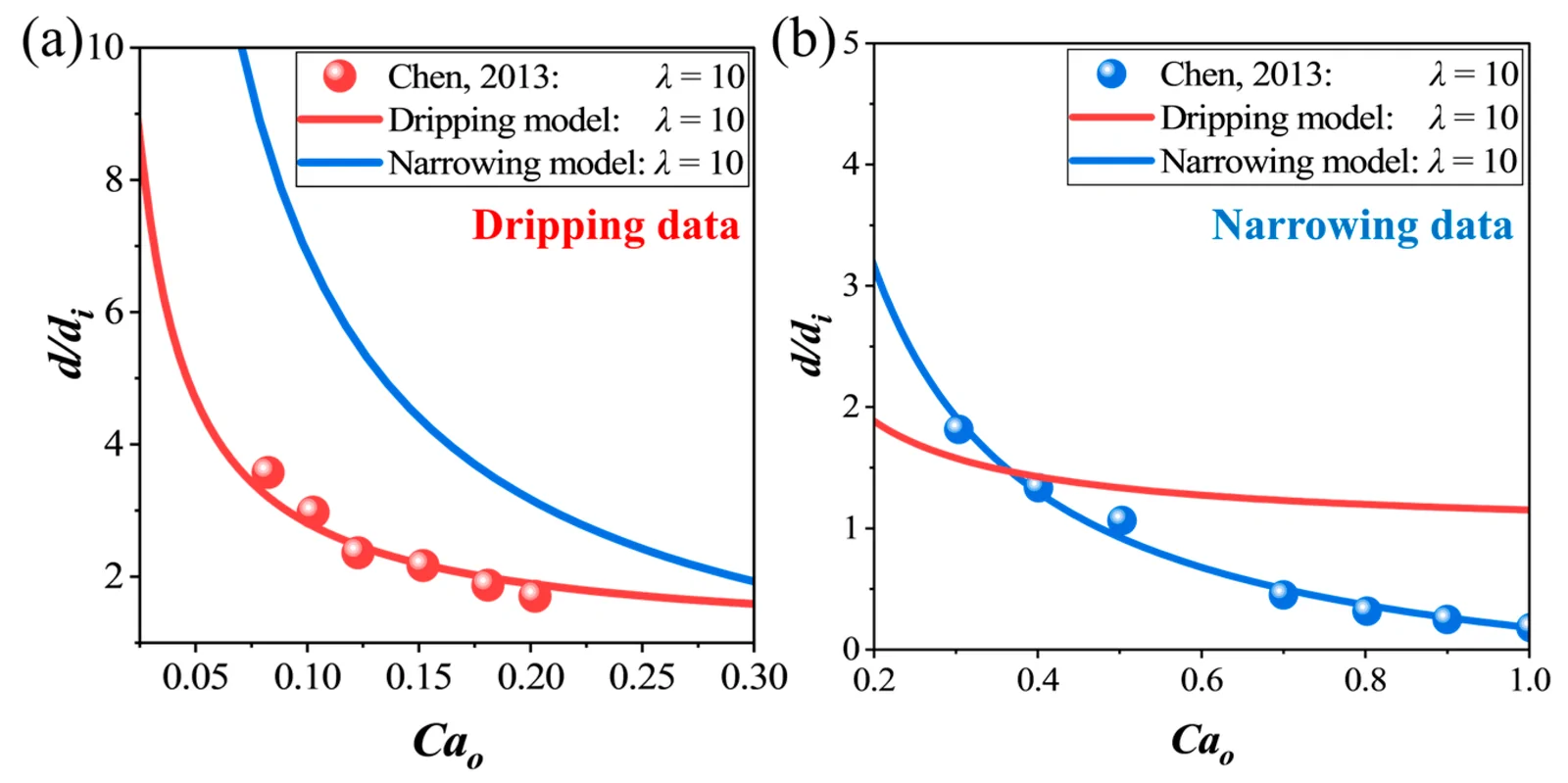
Cross-validation of the regime-specific models against the experimental data of Chen et al. (2013) [34] at λ=10 : (**a**) dripping data predicted using the dripping and narrowing-jetting models; (**b**) narrowing-jetting data predicted using the dripping and narrowing-jetting models.

## Data Availability

The raw data supporting the conclusions of this article will be made available by the authors on request.

## Appendix A

Figure A1 presents a cross-validation of the dripping and narrowing-jetting models using the experimental data of Chen et al. (2013) [34] at λ=10. For the dripping data, the dripping model reproduces the experimental trend well, whereas the narrowing-jetting model substantially overpredicts the droplet size. Conversely, for the narrowing-jetting data, the narrowing-jetting model follows the experimental measurements closely, while the dripping model increasingly deviates as $Ca_{o}$ increases. These results confirm that the two regimes require separate formulations because of their distinct droplet-formation mechanisms.
